# Restoration of a Mediterranean forest after a fire: bioremediation and rhizoremediation field-scale trial

**DOI:** 10.1111/1751-7915.12138

**Published:** 2014-07-31

**Authors:** Paloma Pizarro-Tobías, Matilde Fernández, José Luis Niqui, Jennifer Solano, Estrella Duque, Juan-Luis Ramos, Amalia Roca

**Affiliations:** 1 Bio-Ilíberis R&DPolígono Industrial Juncaril, Peligros, Granada, 18210, Spain; 2 Estación Experimental del Zaidín-CSICGranada, Granada, 18008, Spain

## Abstract

Forest fires pose a serious threat to countries in the Mediterranean basin, often razing large areas of land each year. After fires, soils are more likely to erode and resilience is inhibited in part by the toxic aromatic hydrocarbons produced during the combustion of cellulose and lignins. In this study, we explored the use of bioremediation and rhizoremediation techniques for soil restoration in a field-scale trial in a protected Mediterranean ecosystem after a controlled fire. Our bioremediation strategy combined the use of *Pseudomonas putida* strains, indigenous culturable microbes and annual grasses. After 8 months of monitoring soil quality parameters, including the removal of monoaromatic and polycyclic aromatic hydrocarbons as well as vegetation cover, we found that the site had returned to pre-fire status. Microbial population analysis revealed that fires induced changes in the indigenous microbiota and that rhizoremediation favours the recovery of soil microbiota in time. The results obtained in this study indicate that the rhizoremediation strategy could be presented as a viable and cost-effective alternative for the treatment of ecosystems affected by fires.

## Introduction

During high temperatures and lack of rainfall, forest fires represent the most frequent perturbation within Mediterranean ecosystems (Hernández *et al*., 1997; Vila-Escalé *et al*., 2007). Loss of forest mass is an extended concern throughout the Mediterranean basin; in 2012 almost 200 000 hectares (Ha) of forests were affected by fire in Spain. This amount was three times the land area affected in 2011, making it one of the most devastating years for forest biomass in the Iberian Peninsula (https://magrama.gob.es/es/desarrollo-rural/temas/politica-forestal/incendios-forestales/lucha.aspx). While drought and heat are natural causes of wildfires, many occur due to incorrect agricultural management, negligence or as the result of economic interests (Olivella *et al*., 2006; Vergnoux *et al*., 2011).

Fire-induced perturbations comprise changes in the physical, mineralogical, chemical and biological properties of soil (Certini, 2005), with levels of severity depending on the intensity and duration of combustion (Campbell *et al*., 1994; Franklin *et al*., 1997; DeBano *et al*., 1998). The immediate effects of fire on soil include: (i) the incineration of associated vegetation cover, which changes nutrient availability and surface organic matter content (Vázquez *et al*., 1993), (ii) a significant decrease in microbial cell density per gram of soil (DeBano *et al*., 1998; Certini, 2005), (iii) compositional changes in soil microbial populations (Torres and Honrubia, 1997; Smith *et al*., 2008), (iv) reduction of water infiltration and rainfall retention, which is required to support plants and, thus, important for resisting erosion (DeBano, 2000; González-Pérez *et al*., 2004) and (v) the release of several pyrolytic substances as polycyclic aromatic hydrocarbons (PAHs), which are toxic and have a tendency to accumulate in tissues (Vila-Escalé *et al*., 2007). Soil dynamics depend not only on physicochemical properties, but also on microbiological health because the return of vegetation after a fire is directly impacted by the metabolic activity of microorganisms, which facilitate nutrient cycling (Certini, 2005).

Technology for the remediation of PAH-polluted sites has traditionally been centred on physicochemical treatments (Fernández *et al*., 2012); however, more recently, the use of microorganisms for *in situ* degradation of pollutants has gained popularity as a bioremediation process (Kuiper *et al*., 2004; Segura *et al*., 2009). In these processes, either native degraders or exogenous microorganisms with appropriate metabolic traits are used. When indigenous microbes are used, the process is known as bioaugmentation (Andreoni *et al*., 2004; Segura *et al*., 2009).

Due to the slow natural recovery of soil after a perturbation, bioremediation techniques have been developed combining microorganisms with plants to accelerate the recovery of soil properties, increase microbial biomass and accelerate plant recolonization. The general process is referred to as phytoremediation, whereas the process is known as rhizoremediation when plants with root-associated microorganisms are used (Kuiper *et al*., 2001; 2004; Wood, 2008; Segura *et al*., 2009; Segura and Ramos, 2012). Hence, in a broad sense, the term bioremediation encompasses rhizoremediation; however, in this article, we will refer to the use of microorganisms alone (without plants) as ‘bioremediation’ to distinguish it from rhizoremediation (joint plant-microbe processes).

There have been few studies that tackle field-scale bioremediation and/or rhizoremediation assays without using physicochemical treatments (Huang *et al*., 2004; Bamforth and Singleton, 2005); in addition, these technologies had not been tested in ecosystems affected by fire, emphasizing the importance of results of the current study. The aims of this current field-scale study were to assess bioremediation and rhizoremediation techniques for the recovery of soil health to pre-fire levels. The chosen methods involved the use of rapidly growing pasture seeds to curb erosion; together with the addition of plant growth-promoting rhizobacteria (PGPR) with biodegradative properties to facilitate the degradation of PAHs, to promote seed germination, plant recolonization and vegetation growth. The results suggest that rhizoremediation was an effective and inocuous treatment to be used in the restoration of Mediterranean ecosystems.

## Results

### Bacterial survival

**Fig 1:**
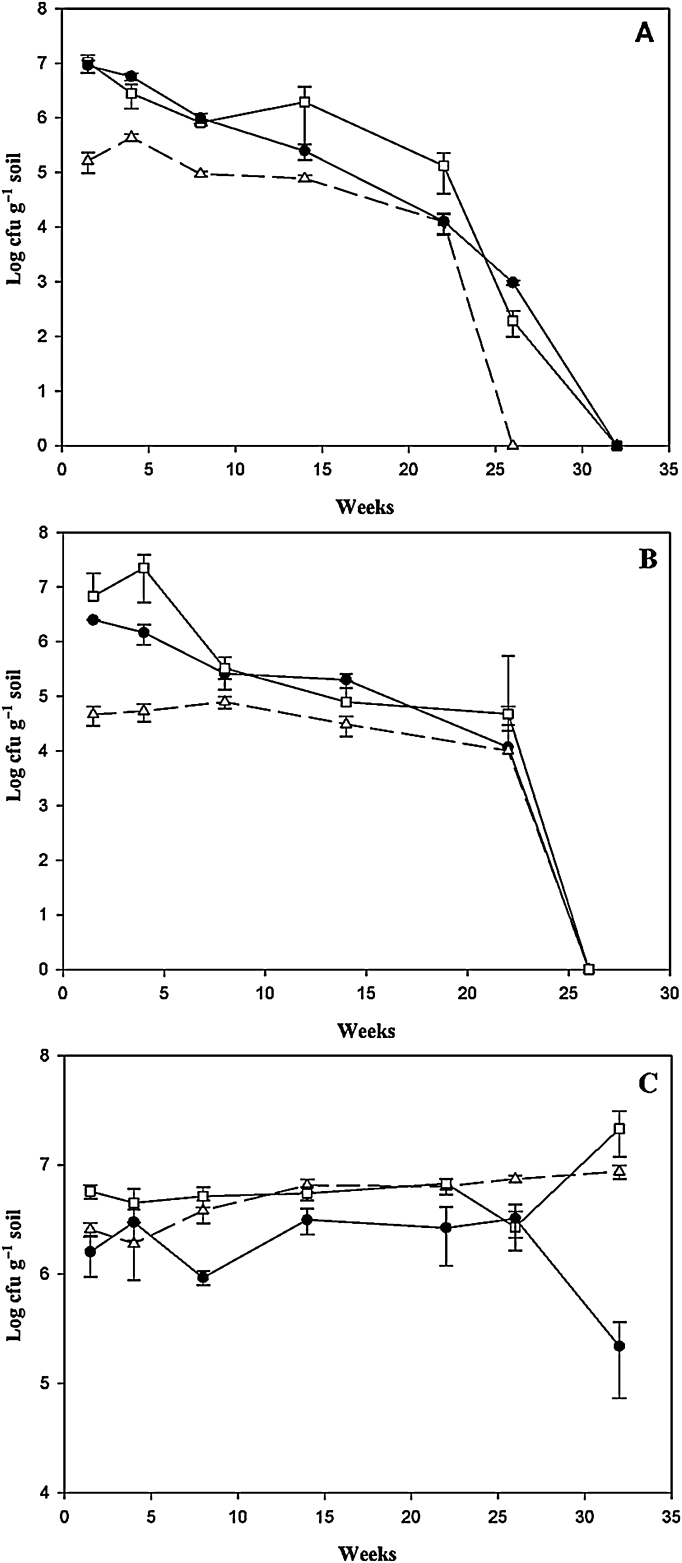
Viable *P. putida* BIRD-1 (A), *P. putida* KT2440 (B) and indigenous microbial consortium (C) in rhizosphere of introduced plants in pristine soil (Treated) (filled circle), in the rhizosphere of introduced plants in burnt soil (Rhizoremediation) (square) and in burnt bulk soil (Bioremediation) (triangle). Data showed as mean (*n* = 3) and error bars refer to standard deviations.

The survival of the introduced microbial consortium (*Pseudomonas putida* strains and indigenous culturable bacteria with biodegradation potential) in burnt bulk soil (Bioremediation treatment), rhizosphere of introduced plants in burnt soil (Rhizoremediation treatment) and in the rhizosphere of plants in pristine soil (Treated control) (Table 1, Fig. 1) was determined by plate counting on selective media; besides, survival of introduced *P. putida* strains was verified by either polymerase chain reaction (PCR) or colony hybridization. The two *P. putida* strains, *P. putida* BIRD-1 and KT2440, that were introduced in soil were able to survive and maintained a population size around 10^6^ colony-forming unit (cfu) g^−1^ soil (from 6.7 × 10^6^ ± 1.1 × 10^6^ to 5.7 × 10^5^ ± 1.7 × 10^4^ cfu g^−1^ soil) for about six months (26 weeks), and then dropped below detection limits during the aestival season (Fig. 1A and B). No significant differences (*P* > 0.05) were detected in the survival of these strains the rhizosphere of plants in the rhizoremediation treatment compared to the treated pristine treatment in most of the sampling times during the first 24 weeks (6 months). Cell densities of these strains in burnt bulk soil (Bioremediation treatment), were significantly lower (*P*  < 0.05) (two orders of magnitude) than in the rhizosphere of plants, whether in burnt or pristine soil. This indicates that survival improved when the strains were associated to plants, especially in the case of *P. putida* BIRD-1 that was detected for seven months in rhizospheric soil.

**Table 1:** Composition of treatments and strains applied to burnt and pristine soil

Burnt soil			
Treatment	Composition	Microorganisms applied	Plant seeds mixture
Control	Untreated bare soil	None	None
Plants control	Non-inoculated plants	None	Peat
			AVEXIII®
			*Trifolium repens*
Bioremediation	Microbial consortium	*P. putida* BIRD-1 (pWW0)	Peat
		*P. putida* KT2440 (pWW0)	
		Indigenous bacterial consortium	
Rhizoremediation	Plants and microbial consortium	*P. putida* BIRD-1 (pWW0)	Peat
		*P. putida* KT2440 (pWW0)	AVEXIII®
		Indigenous bacterial consortium	*Trifolium repens*

**Pristine soil**			
**Treatment**	**Composition**	**Microorganisms applied**	**Plant seeds mixture**
Control	Untreated	None	None
Treated	Plants and microbial consortium	*P. putida* BIRD-1 (pWW0)	Peat
		*P. putida* KT2440 (pWW0)	AVEXIII®
		Indigenous bacterial consortium	*Trifolium repens*

As expected, the culturable indigenous hydrocarbon-degrading microbial consortium, monitored by plate counting with diesel fuel as a sole carbon source (Fig. 1C), revealed a steady population size around 10^6^ cfu g^−1^ soil (from 1.6 × 10^6^ ± 7.9 × 10^5^ to 8.6 ×1 0^6^ ± 1.6 × 10^6^ cfu g^−1^ soil). These population levels remained quite constant until the end of the study, showing no significant differences (*P* > 0.05) regardless of whether the soil was pristine or burnt or whether exogenous *P. putida* strains and/or plants were present at most sampling times (Fig. 1C), except at the end of the study (32 weeks) in which cell densities in pristine soil dropped one order of magnitude.

### Metagenomic analysis of soil microbial population

In order to determine the consequences of fire on soil microbiota, as well as to study the effect of rhizoremediation treatments over the spectrum of indigenous microbial populations, a metagenomic analysis of 16S RNA for bacterial biodiversity was carried out at month 1 (autumn, November 2008) and 6 months after the beginning of the trial (spring, April 2009) for Control burnt soil (bulk), Control pristine soil (rhizospheric) and soil undergoing Rhizoremediation (rhizospheric).

Rarefaction analyses were performed to compare bacterial richness among pristine, burnt and soil undergoing rhizoremediation. Analyses were based on a minimum of 125 sequences and the number of operational taxonomic units (OTUs) were estimated using a cut-off of 97% for sequence similarity a generally accepted level for comparative analysis of whole and partial 16S rRNA sequences (Konstantinidis *et al*., 2006). The rarefaction curve (Supporting Information Fig. S2) showed a similar number of OTUs in all soil samples, indicating similar bacterial richness.

**Fig 2:**
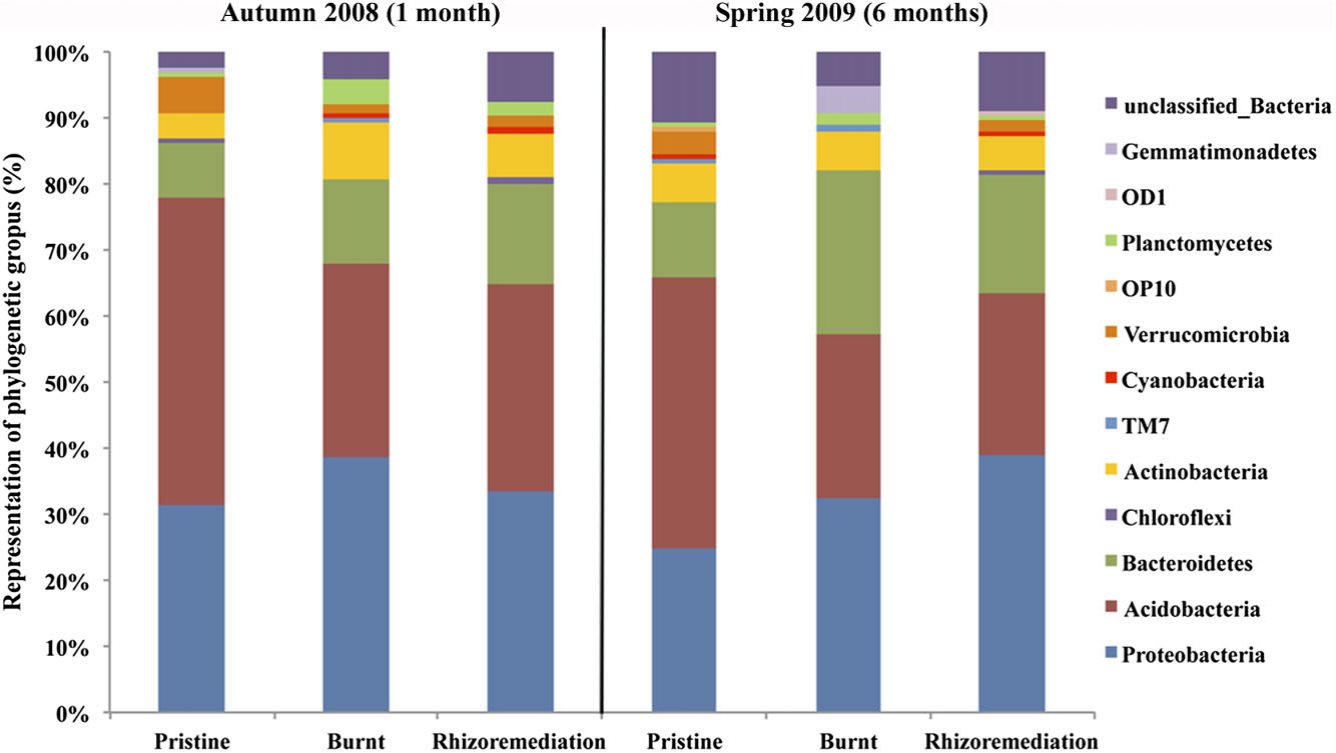
Relative abundance of phylogenetic groups at phylum level based on 16S rRNA genes. Pristine soil, bulk burnt soil and rhizoremediation treatment at two different times of the assay 1 month (autumn) and 6 months (spring).

The analysis of relative abundance at phylum level (Fig. 2) showed changes in the bacterial community distribution and proportion. *Proteobacteria*, *Acidobacteria* and *Bacteriodetes* were the predominant phyla in all the cases we studied; combined, these three phyla constituted 80% of the total. Specifically, *Acidobacteria*, which was the prevailing phylum in pristine soil and 46% of the total, experienced a remarkable population reduction to 29% of the total in burnt soil. In contrast to *Acidobacteria*, an increase in the proportion of *Proteobacteria* (from 31.3% to 38.6%) and *Bacteriodetes* (from 8.4% to 12.9%) was observed in Control burnt soil.

Less common phyla exhibited significant changes in their relative abundance: *Verrucomicrobia*, which made up 5.3% of the total in pristine soil, dropped to 1.4% in Control burnt soil and *Chloroflexi* and *Gemmatimonadetes* dropped below detection limits; nevertheless, the proportion of these phyla was less than 1% in pristine soil (0.8%). On the other hand, *Actinobacteria* was more abundant in burnt (8.6%) than in pristine samples (3.8%) at the first sampling time point; similarly, *Gemmatimonadetes* was also more predominant in burnt (4.2%) than in pristine (undetectable) soil at the last sampling time point. No bacterial phylum was reduced exclusively in soil under Rhizoremediation treatment.

**Fig 3:**
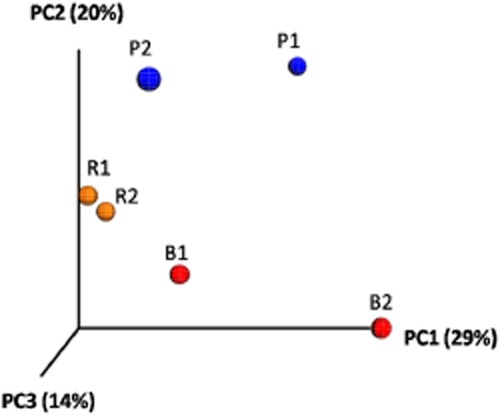
Principal coordinate analysis (PCoA) constructed using FASTUNIFRAC software (Hamady *et al*., 2010) to compare sample groups from phylogenetic distance-matrix, determining similarity between or differences among pristine soil (P), bulk burnt soil (B) and rhizoremediation treatment (R) at two different times of the assay 1 month (autumn) and 6 months (spring). 3D image obtained through EMPeror software (Vazquez-Baeza *et al*., 2013).

A principal coordinate analysis (PCoA) was performed to compare genetic distance matrix between groups (Fig. 3). Statistical differences were observed in the phylogenetic composition of microbial populations between burnt and pristine soil, with intermediate values observed for sites that were treated using Rhizoremediation. Moreover, Control burnt and Control pristine soil microbial populations showed season-induced variations while the Rhizoremediation ones remained quite unaltered through sampling times.

### Soil hydrocarbon monitoring

At the beginning of the assay, a number of pyrolytic hydrocarbons such as monoaromatic hydrocarbons (BTEX: benzene, toluene, ethylbenzene and xylene) and PAHs that had been generated during the fire were detected (Table 2). An average concentration of 149.7 ± 17.5 μg kg^−1^ soil for BTEX and 398.0 ± 32.4 μg kg^−1^ soils for PAHs were measured in burnt plots; accordingly, these compounds were below detection limits in Control pristine soil (data not shown).

**Table 2:** Concentration of pyrolytic hydrocarbons (μg per kg of soil) generated after fire (Initial concentration, *n* = 20), versus the concentration of the same substances measured after 2 months of treatment (*n* = 5) (Control burnt, bioremediation, untreated plants and rhizoremediation treatments). Results are expressed as the mean and standard deviation. ND means below detection limits (10 μg kg^−1^ and 0.5 μg kg^−1^ for BTEX and PAHs respectively)

				Two months after the outset of the study (December 2008)			
Pyrolitic substances	Rings	Compounds	Initial concentration (October 2008) (μg kg^−1^)	Control burnt (μg kg^−1^)	Plants control (μg kg^−1^)	Bioremediation (μg kg^−1^)	Rhizoremediation (μg kg^−1^)
BTEX	1	Benzene	38.1 ± 5.3	ND	ND	ND	ND
		Toluene	62.4 ± 7.2	ND	ND	ND	ND
		Ethylbenzene	17.0 ± 0.9	ND	ND	ND	ND
		Xylene	32.2 ± 4.1	ND	ND	ND	ND
*TOTAL BTEX*			149.7 ± 17.5	ND	ND	ND	ND
PAHs	2	Naphthalene	118.0 ± 8.9	31.6 ± 2.3	34.0 ± 2.6	43.2 ± 2.9	26.8 ± 1.3
	3	Acenaphthene	ND	ND	ND	ND	ND
		Fluorene	48.3 ± 3.0	1.9 ± 0.7	1.7 ± 0.4	1.7 ± 0.1	ND
		Phenanthrene	121.0 ± 9.9	26.9 ± 3.1	15 ± 2.0	11.5 ± 2.4	9.5 ± 0.7
		Anthracene	1.6 ± 0.2	1.6 ± 0.3	ND	ND	ND
	4	Fluoranthene	26.0 ± 3.1	6.4 ± 1.3	ND	ND	ND
		Pyrene	29.6 ± 2.0	8.4 ± 2.1	1.7 ± 0.1	1.0 ± 0.3	0.5 ± 0.1
		Benzo(a)Anthracene	12.5 ± 1.1	1.2 ± 0.2	1.5 ± 0.3	ND	ND
		Crysene	8.6 ± 0.3	6.9 ± 2.1	0.3 ± 0.1	ND	ND
	5	Benzo(b)Fluoranthene	1.5 ± 0.3	2.0 ± 0.3	ND	ND	ND
		Benzo(k)Fluoranthene	0.8 ± 0.1	0.9 ± 0.1	ND	ND	ND
		Benzo(a)Pyrene	17 ± 0.6	1.9 ± 0.1	0.6 ± 0.1	ND	ND
	6	Dibenzo(a,h)Anthracene	8.1 ± 1.7	ND	ND	ND	ND
		Benzo(g,h,i)Perylene	2.0 ± 0.7	3.9 ± 0.4	ND	ND	ND
		Indene	3.0 ± 0.4	ND	ND	ND	ND
*TOTAL PAHs*			398.0 ± 32.3	93.6 ± 10.7	54.8 ± 5.6	57.4 ± 5.7	36.8 ± 2.1

After 2 months of treatment, BTEX concentrations in the burnt plot dropped below detection limits. Low molecular PAHs, comprising two to four carbon rings, made up the main fraction of the total PAHs (≈ 95%) in burnt plots. The dominant PAH compounds were naphthalene and phenanthrene, which each made up ≈ 30% of the total. Two months after the beginning the assay, the concentration of total PAHs on Plants control and bioremediation treatments was a 40% lower than in Control burnt soil, being up to a 60% lower were soil had been undergoing Rhizoremediation. At this point, most of the initially measured compounds were still found in the Control burnt soil, whereas in the Rhizoremediation treatment only naphthalene, phenanthrene and pyrene were remaining.

### Soil quality indicators

Soil quality indicators, such as soil pH and enzyme activity, can be used as an indirect measure of soil quality changes or as indexes of soil disturbance or restoration (Karaca *et al*., 2011).

#### pH

**Fig 4:**
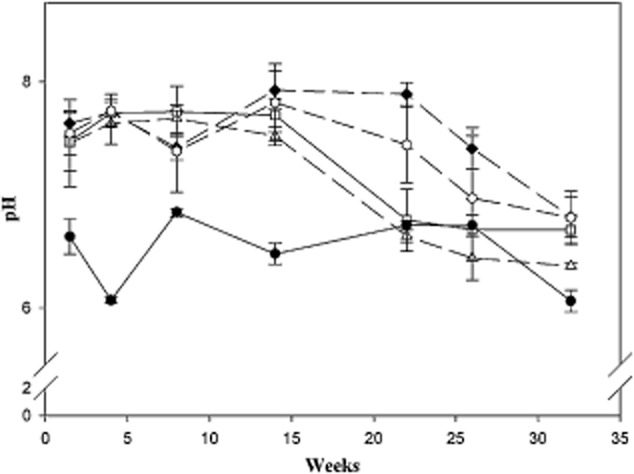
pH measurements performed along the study during 8 months, on pristine soil (P) (filled circle), burnt bulk soil (C) (filled diamond), bioremediation (B) (triangle) and rhizoremediation (R) (square), and plants control (CP) (hexagon). Data are shown as mean (*n* = 3) and error bars refer to standard deviations.

A significant soil alkalynization (*P* < 0.05) was observed in burnt soil (pH 7.63 ± 0.11) versus control pristine soil (pH 6.53 ± 0.15) (Fig. 4). pH among burnt soil treatments (Control bulk soil, Plants control, Bioremediation and Rhizoremediation) showed no significant differences (*P* > 0.05) in the first 4 months of treatment. Then, all treatments experienced a progressive decrease of pH. Nevertheless, both soils treated with Bioremediation and Rhizoremediation treatments experienced a decrease in pH (6.63 ± 0.05 and 6.73 ± 0.04, respectively), reaching Control pristine soil pH levels (6.70 ± 0.04) after 5 months (22 weeks).

#### Enzymatic activity assays

**Fig 5:**
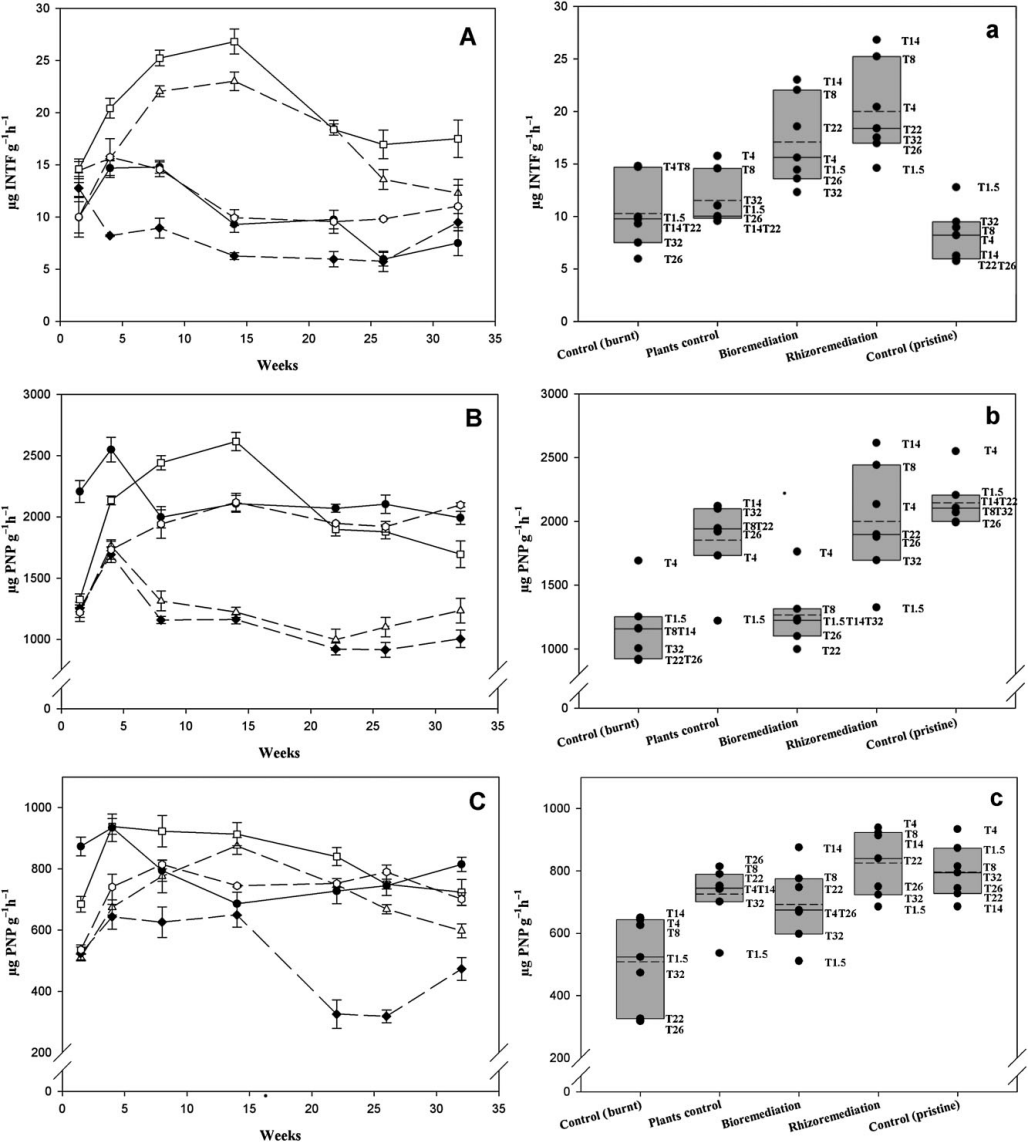
Enzymatic activities measurements performed along the study during 8 months. (Aa) show dehydrogenase activity, (Bb) show phosphatase activity and (Cc) shows β-glucosidase activity assessed on Control pristine soil (filled circle), Control burnt soil (filled diamond), Bioremediation (triangle) and Rhizoremediation (square), and plants control (circle). A–C data are shown as mean (*n* = 3) and error bars refer to standard deviations. a–c data are shown as mean (*n* = 3), vertical boxes show the median (solid line), mean (dash line) and the 5th/95th percentiles.

In this study, the evolution of three different enzymatic activities was monitored (Fig. 5): (i) β–glucosidases, which are involved in the saccharification of cellulose, (ii) dehydrogenases, which take part in reactions involved in energy transfer in microbial metabolism reactions and (iii) phosphatases, which hydrolyze organic phosphorous compounds to different forms of inorganic phosphorous (Karaca *et al*., 2011). β–glucosidase and phosphatase are directly involved in C and P cycles respectively (Bandick and Dick, 1999).

Dehydrogenase activity (DHA) was measured as the amount of iodonitrotetrazolium violet-formazan (INTF) released from 1 g soil after 20 h incubation in the dark (Fig. 5A). DHA monitoring showed that levels in all treatments varied in the range between 10.0 ± 1.5 to 14.6 ± 0.7 μg INTF g^−1^h^−1^, whether burnt or pristine soil at the beginning of the assay. Bioremediation and Rhizoremediation treatments steadily raised DHA levels for about 15 weeks, leading to total increases of 2.5-fold (23.0 ± 0.9 μg INTF g^−1^h^−1^) and 3-fold (26.8 ± 1.2 μg INTF g^−1^h^−1^), respectively, over Control burnt soil (9.3 ± 0.8 μg INTF g^−1^h^−1^). All non-inoculated treatments (Control pristine soil, Control burnt soil and Control plants) remained with lower DHA activity, below 15.0 μg INTFg^−1^h^−1^, along the assay; as can be ascertained from Fig. 5A, treatments grouped in inoculated (Bioremediarion and Rhizoremediation) and non-inoculated (Control pristine and burnt soil and Plants control) along the assay. No differences (*P* > 0.05) were found between plants control and pristine soil at most sampling times. At the end of the assay (July 2009), DHA in Rhizoremediation treatment remained significantly higher (*P* < 0.05) (17.5 ± 1.8 μg INTFg^−1^h^−1^) when compared with the rest of the treatments (7.5 ± 1.2 μg INTFg^−1^h^−1^ in Control burnt soil; 11.3 ± 2.1 μg INTFg^−1^h^−1^ in Plants control; 12.3 ± 1.3 μg INTFg^−1^h^−1^ in Bioremediation and 9.5 ± 0.8 μg INTFg^−1^h^−1^ in Control pristine soil).

Phosphatase activity (Fig. 5B) was found to be 2210 ± 90 μg p-Nitrophenol (PNP) g^−1^h^−1^ in Control pristine soil at the beginning of the assay, whereas activity in the burnt plot was below 1325 ± 45 μg PNP g^−1^h^−1^. Fire led to a significant decrease (*P* < 0.05) in activity by about 40%. After 8 weeks of treatment, phosphatase activity in the Plants control treatment was found to reach Control pristine soil levels (2000 ± 90 μg PNP g^−1^h^−1^), and also in Rhizoremediation treatments, where phosphatase activity had increased up to 2440 ± 60 μg PNP g^−1^h^−1^, doubling its initial values. Along the assay, phosphatase activity was higher in treatments with associated plants (Control pristine soil, Plants control and Rhizoremediation) than in those without (Control burnt soil and Bioremediation) (Fig. 5C). Phosphatase activity in Control burnt soil and in Bioremediation treatment remained unaltered between 1000 and 1500 μg PNP g^−1^h^−1^ along the assay. At the end of the trial, measurements in treatments where plants had been introduced (Rhizoremediation and Plants control) were found to still be higher than those in bulk soil (Control burnt soil and Bioremediation).

At the beginning of the assay, β–glucosidase activity (Fig. 5C) was also affected by fire, significantly decreasing (*P* < 0.05) by 20 (Rhizoremediation) and about a 40% (the rest of treatments), when compared with Control pristine soil. These levels were restored after 4 weeks in Rhizoremediation treatment (fluctuating between 750 ± 40 and 940 ± 30 μg PNP g^−1^h^−1^), whereas Bioremediation required 3 months to reach Rhizoremediation parameters. Along the assay, β–glucosidase activity was found to be at Control pristine levels in all treatments, except for Control burnt soil (Fig. 5C). At the end of the trial, β–glucosidase activity was higher in all treatments when compared with Control burnt soil (475 ± 40 μg PNP g^−1^h^−1^), especially in Rhizoremediation treatment (725 ± 40 μg PNP g^−1^h^−1^).

### Plant fitness and effect on the landscape

To evaluate plant fitness, we measured weight, length of roots and aerial parts of plants between the various conditions (Table 3). Introduced plants (Avex III® and Clover) used on this study showed increased size and dry weight when inoculated with bacteria (*P* < 0.05). Length increases were 25% for clover and 58% for Avex III**®** plants (*P* < 0.05); average dry weight increases were 43% for clover and 67% for Avex III**®** (*P* < 0.05), after fourteen weeks of treatment (February 2009).

**Table 3:** Introduced plants measurements after 14 weeks of the beginning of the assay. Results are expressed as the mean (*n* = 3) and standard deviation. For each introduced plants (Avex III® and Clover) and each parameter, different letters mean statistical differences according to the Tukey test (*P* < 0.05)

	Avex III®		Clover	
	Control plants	Rhizoremediation	Control plants	Rhizoremediation
Length (cm)	13.84 ± 0.99b	21.97 ± 1.65a	8.28 ± 1.04B	10.54 ± 0.68A
Fresh weight (g)	3.56 ± 0.69b	6.10 ± 0.95a	5.14 ± 1.62B	9.02 ± 1.29A
Dry weight (g)	1.19 ± 0.13b	1.98 ± 0.31a	2.04 ± 0.12B	2.92 ± 0.17A

**Fig 6:**
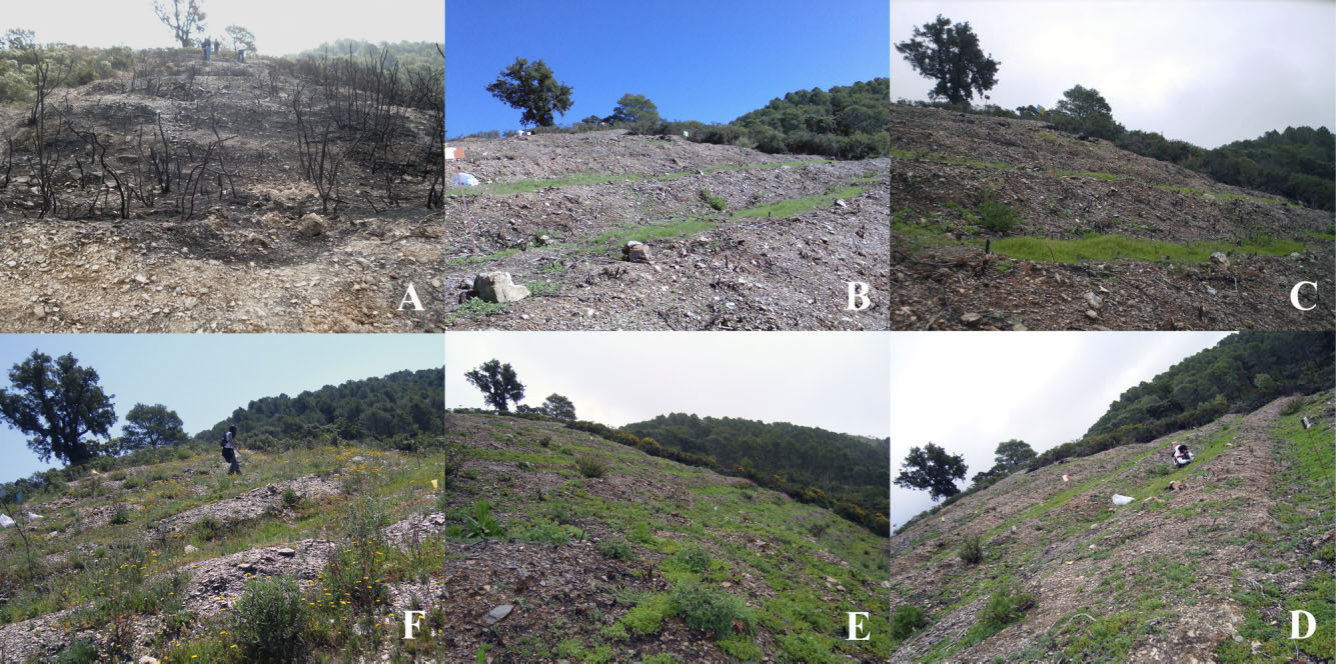
General evolution of the treated burnt parcel along the study (8 months, from October 2008 to June 2009). Clockwise: before treatments setting (October 2008) (A), 4 weeks after beginning of the trial (November 2008) (B), 8 weeks (December 2008) (C), 14 weeks (February 2009) (D), 22 weeks (April 2009) (E) and 32 weeks (July 2009) (F).

It should be noted that 4 weeks after the beginning of the assay, the plant growth promoting (PGP) effect of the artificial consortium over introduced vegetation development and soil coverage could be perceived in rhizoremediation versus non-inoculated plant subplots (Supporting Information Fig. S3). Finally, the visual impact of bioremediation and rhizoremediation processes on the landscape was documented by a series of photographs (Fig. 6).

## Discussion

### Bacterial performance in bioremediation and rhizoremediation technologies

The ability to survive in harsh, polluted environments is one of the key requirements of the microorganisms selected for bioremediation processes. For survival, microorganisms need to show evidence of: (i) enhanced adaptation to the particular environment undergoing remediation and (ii) mechanisms to overcome the deleterious effects caused by the pollutant(s).

*Pseudomonas putida* strains are excellent candidates for soil restoration, especially in rhizoremediation processes because they are good rhizosphere colonizers (Molina *et al*., 2000; Lugtenberg *et al*., 2001; Ramos-González *et al*., 2005; Matilla *et al*., 2011; Wu *et al*., 2011; Roca *et al*., 2013) and metabolically versatile (Palleroni, 1992; 2010). Apart from these important traits, our results revealed that the *Pseudomona*s strains used in this study can overcome the toxic effects of hydrocarbon compounds produced during the combustion of organic matter. Microbial tolerance to hydrocarbons has been linked to the strains' ability to degrade these compounds (Park *et al*., 2004); nevertheless, tolerance is a relatively complex process involving the activation of extrusion mechanisms, the establishment of oxidative stress responses and an overall fitness programme (Silby *et al*., 2011; Krell *et al*., 2012). *Pseudomonas putida* KT2440 and BIRD-1 lack the metabolic potential for hydrocarbon degradation; however, the transfer of the pWW0 plasmid endows them with the ability to degrade some BTEX compounds.

From the multiple parameters presented in this field assay, only drought conditions showed a clear negative effect on introduced *P. putida* strains; in fact, the decrease in added *Pseudomonas* populations concurred with the wilting of plants during the summer season when lack of precipitation and high temperatures converge. Monitoring of native culturable hydrocarbon degraders showed that these populations remained unaltered through fires and landscape and climate variations, whereas introduced strains failed to survive through climate variations. As Mediterranean ecosystems are constantly affected by fires (Hernández *et al*., 1997; Vila-Escalé *et al*., 2007), indigenous microbial adaptation to this environment seems to have occurred (Fonturbel *et al*., 1995; Choromanska and DeLuca, 2001; Smith *et al*., 2008).

### Impact of fire and rhizoremediation treatments on soil microbial populations

Fires have been studied for their ability to change soil properties (Vázquez *et al*., 1993; Certini, 2005) and disrupt indigenous microbial populations (Torres and Honrubia, 1997; DeBano *et al*., 1998; Certini, 2005; Smith *et al*., 2008). We carried out biodiversity analysis, which corroborated the resilience of native Mediterranean microbiota because only changes in the bacterial community distribution were observed, with no population loss (Supporting Information Fig. S2). These findings reinforce the hypothesis that indigenous microorganisms in Mediterranean ecosystems exhibit a high level of adaptation to fires (Fonturbel *et al*., 1995; Hernández *et al*., 1997; Choromanska and DeLuca, 2001; Vila-Escalé *et al*., 2007; Smith *et al*., 2008). Nevertheless, changes in the composition of microbial populations had been previously observed in connection with soil deterioration/pollution generated by fire: (i) increasing soil pH values because of soil organic matter denaturation lead to a decrease in *Acidobacteria* and to an increase in *Bacteroidetes* populations (Certini, 2005; Smith *et al*., 2008; Lauber *et al*., 2009), (ii) the ratio of *Proteobacteria* increased in burnt plots, a phylum where strains with enhanced abilities for PAH metabolism can be found (Mueller *et al*., 1997; Watanabe *et al*., 2001), whereas microbial populations that lack this ability do not proliferate in these soils because of the selective pressure these compounds may exert (Martínez *et al*., 2000) and (iii) the relative abundance of *Gemmatimonadetes* is increased in burnt soils and is also modulated by soil aridness (DeBruyn *et al*., 2011), pH (Lauber *et al*., 2009) and the presence of pyrogenic carbon (Khodadad *et al*., 2011).

The Rhizoremediation treatment may introduce factors that can affect the structure of soil microbial communities, including the introduction of exogenous bacteria, the introduction of exogenous vegetal species and enrichment in indigenous culturable bacteria. Nevertheless, as we mentioned above, the relative abundance of most of the bacterial groups in soils undergoing rhizoremediation was intermediate between burnt and pristine forest soil, which suggests that the main perturbation on indigenous microbial populations was fire. This also suggests that the Rhizoremediation treatment tended to restore the original structure of microbial communities, probably due to the observed restoration of soil characteristics such as pH, vegetal cover and decreases in PAHs.

### Rhizoremediation enhances hydrocarbon degradation

One of the main consequences observed after a fire is the generation of new, toxic and recalcitrant forms of carbon (González-Pérez *et al*., 2004), such as PAHs (Vila-Escalé *et al*., 2007), as well as associated volatile hydrocarbons such as BTEX (Bamforth and Singleton, 2005). The PAH profiles observed in the current study comprised 60% of naphthalene and phenanthrene, which is consistent with reported profiles corresponding to wood combustion (Xu *et al*., 2006; Kim *et al*., 2011) and, in particular, to pine needles and wood (Conde *et al*., 2005).

The lower complexity of these monoaromatic chemical structures (Bamforth and Singleton, 2005), as well as the increase in the proportion of bacterial populations with degrading potential, rapidly cleared these compounds from burnt soils, regardless of applied treatment. Furthermore, bio-attenuation mediated decreases in PAHs in untreated soil because of the presence of native degraders.

Nevertheless, Rhizoremediation treatments promoted the almost complete removal of heavier pyrolytic hydrocarbons in a relatively short time, which indicates that the combination of microorganisms, introduced PGPR, native degraders and plants was the most effective method for remediation. Because the introduced *P. putida* strains were not PAHs degraders, native microorganisms played a central role in PAH elimination. Because native populations were not significantly increased, it appears that the rhizosphere exerts a direct effect on stimulating the expression and/or activity of bacterial catabolic pathways, as was proved recently for naphthalene degradation by *P. putida* (Fernández *et al*., 2012).

### Soil quality parameters indicated best restoration through rhizoremediation

The first, and most noticeable, consequence of a forest fire is the black ash coat (Knicker, 2007) generated by the combustion of the vegetation layer, which leads to the release of cations (Certini, 2005; Smith *et al*., 2008). This explains the increase in soil pH observed after burning (Fig. 4). Because changes in pH can negatively affect microbial populations and their ability to degrade toxic compounds (Leahy and Colwell, 1990), the stabilization of pH to pre-fire levels is vital in remediation strategies. Our study revealed that Bioremediation and Rhizoremediation treatments were equally effective at restoring pH levels to pre-fire levels. This is also apparent, as non-inoculated plants were less capable of restoring pH levels versus inoculated plants. This soil acidification could be ascribed to the increase of soil microbial activity, as introduced an indigenous microorganisms produce acids and enzymes to solubilize soil nutrients, increasing their availability for plants and microorganisms alike. The same effect takes place for available P (Supporting Information Table S1), which slightly increases after fire and achieved maximal concentration in soil after treatment. *Pseudomonas putida* BIRD-1, one of the microorganisms used in this study, is capable of producing several enzymes and acids in order to solubilize insoluble phosphates (Roca *et al*., 2013). The use of this bacterium favours the availability of soluble forms of phosphate for plants.

After fire, an increase of organic matter and total N was observed (Supporting Information Table S1), as previously described by Certini (2005). These parameters positively evolve along the treatment, as consequence of introduction of plants in rhizoremediation treatment and the improvement in soil microbial activity. An increase of available K is noticed after fire (Supporting Information Table S1), which is in concordance to parameters described by Khanna and Raison (1986). The proportion of cations (K^+^) decreases along the study as consequence of lixiviation and/or runoff (Certini, 2005).

An increase in salinity was also noticed after fire (Supporting Information Table S1), as described by several authors (Naidu and Srivasuki, 1994; Hernández *et al*., 1997), as consequence of the liberation of organic cations from organic matter. Soil conductivity increased after treatment, as consequence of the liberation of ions and cations due to solubilization of soil nutrients mediated by the increase of microbial activity.

In soil ecotoxicology, soil enzyme activities are used as indexes of soil disturbance or restoration because of their sensitivity to natural and anthropogenic induced stresses. These indexes are easy to measure and can detect the impact of microbial activities on nutrient cycling (Nannipieri *et al*., 2002; Gianfreda *et al*., 2005; Karaca *et al*., 2011). In this study, three different enzymatic activities were monitored (Fig. 5): (i) dehydrogenases, which take part in reactions involved in energy transfer in microbial metabolism (Karaca *et al*., 2011), (ii) phosphatases, which hydrolyze organic phosphorous compounds into different forms of inorganic phosphorous (Karaca *et al*., 2011) and (iii) β-glucosidases, which are involved in the saccharification of cellulose. β-glucosidases and phosphatases are directly involved in C and P cycles respectively (Bandick and Dick, 1999).

Our results support the previous proposal that soil enzymatic activities are reliable indicators of the health and functionality of microorganisms in response to fire stress (Fioretto *et al*., 2005) and that hydrocarbon levels can exert negative effects, to varying degrees, on these activities (Kiss *et al*., 1998). Phosphatase and β-glucosidase activities were, as expected, severely affected by fire (Saa *et al*., 1993; Eivasi and Bayan, 1996; Boerner *et al*., 2000; Boerner and Brinkman, 2003). During the study period, phosphatase activity was clearly related to the presence of vegetation and only reached pristine levels with the Rhizoremediation treatment as a result of the secretion of this enzyme by root exudates (Tarafdar and Claassen, 1988). Changes in phosphatase and β-glucosidase activities were similar, remediation hastened recovery to levels found in pristine soils because of the positive effect of the rhizosphere on microbial activity (Valé *et al*., 2005) and the provision of substrates from rhizodeposition (Morgan and Whipps, 2001) leading to improved enzyme synthesis (Turner *et al*., 2002). In contrast, DHA measurements showed that soil microbiota was not severely affected by fire, as it is a direct indicator of respiration of viable cells (García *et al*., 1997). This could be due to the positive ‘fertilizing effect’ that nutrients from charred necromass provide (Baath and Arnebrant, 1994), as well as to the previously discussed adaptations to fire of native strains. Nevertheless, bioremediation and rhizoremediation treatments showed improvements over untreated plots because of the bioaugmentation of native and added microorganisms, and the development of a vegetation cover.

In all cases, the use of Bioremediation processes enhanced soil quality parameters. Burnt soils reached pristine parameters faster with rhizoremediation providing the most remarkable benefits because of the greater microbial activity provided by the release of enzymes and substrates in root exudates (Badalucco and Kuikman, 2001).

### Landscape restoration

Germination and growth of inoculated plants was rapid in comparison with non-inoculated plants, which were negatively affected by the presence of pyrolytic pollutants. Furthermore, higher degradation rates and microbial activities were observed for inoculated plants. These results emphasize the important link between pollutant removal and the generation of adequate niches for native degraders (Aprill and Sims, 1990; Segura *et al*., 2009; Segura and Ramos, 2012). Furthermore, the use of PGPR has shown to alleviate pollutant-induced plant stress (Qiu *et al*., 1994; Kuiper *et al*., 2001; Zhuang *et al*., 2007). On this issue, *Pseudomonas putida* BIRD-1 presents itself as an interesting strain in Rhizoremediation due to its robust PGP properties (Matilla *et al*., 2011; Roca *et al*., 2013) and proved tolerance to soil hydrocarbons.

The use of PGPR- and native degraders-inoculated pasture plants in this study provided remediation advantages because of (i) the rapid ease of the visual impact through rapidly growing aerial plants, (ii) enhanced soil coverage, (iii) the added substrate and reduced erosion provided by large root surfaces with extensive soil penetration, (iv) the establishment of suitable niches for enhanced degradation processes based on the establishment of microbial consortia and (v) the increase of microbial activity.

## Experimental procedures

### Field experiment site description

The protected Parque Natural de los Montes de Málaga (http://www.juntadeandalucia.es/medioambiente) served as the Mediterranean ecosystem in this study. Located in the south of Spain in the province of Málaga, the park occupies an area close to 5000 Ha (Supporting Information Fig. S1). Average annual temperature is 15°C, and average precipitations are over 600 mm, belonging to the Mediterranean climatic zone. This ecosystem was declared protected in 1989 and comprises *Pinus halepensis*, *Quercus ilex*, *Quercus suber* and *Quercus faginea* forests, accompanied by matching brushwood (*Pistacia lentiscus*, *Rhamnus alaternus*, *Chamaerops humilis*, *Origanum majorana*, *Retama sphaerocarpa*, *Stipa tenacissima*, etc.). The park is also home of endangered species, such as the *Chamaeleo chamaeleon* (chameleon). Other notable vertebrates present in this protected ecosystem comprise the Spanish pond turtle (*Mauremys leprosa*), the endemic iberian worm lizard (*Blanus cinereus*), the short-toed snake eagle (*Circaetus gallicus*), the wild boar (*Sus scrofa*) and an endemic cricket (*Petaloptila malacinata*), among others. Soil geology of this ecosystem consists essentially of sedimentary materials, such as basic sandstones, and slates, having younger deposits of sands, conglomerates, marls and red clays. This mixture of geologic materials composed a high fertile soil that led to a heavy agriculture exploitation, which provoked soil bareness and consequent processes of erosion, leaving some areas of the actual protected ecosystem with undifferentiated lithology, such as the area of our study. The southern Mediterranean region of Spain is heavily affected by fire, the province of Malaga suffered an average of 133 wildfires in the 1992–2002 decade, resulting in an average calcined area of 1631 Ha, concentrated in the aestival season (http://www.magrama.gob.es/es/biodiversidad/temas/incendios-forestales/), so fireguard training and testing of new materials for use in fire extinction is considered to be of great importance (Pausas, 2012). An experimental fire, reaching 450°C on the soil surface [measurement provided by Andalusian Forest Fire Brigade (INFOCA) staff] resulting in the calcination of the brushwood and pine trees (Fig. 6), was induced under the strict supervision of the Andalusian Forest Fire Brigade (INFOCA, http://www.juntadeandalucia.es) in April 2008, allowing us to use it afterwards for this study. Our study was initiated in October 2008.

The burnt plot (N 36° 52.804' - W 004° 21.013') was located on a hill with a high slope that was subdivided into 12 terraces (subplots), each with an area of approximately 100 m^2^, which were cleared of calcined vegetation before setting the assay, including untreated control parcels. A non-burnt plot (pristine soil) was established nearby, which was also divided into terraces (comprising six subplots), for control treatments in pristine soil to be applied in parallel.

### Treatments procedure

Three replicates of the applied treatments (Table 1) were established (3 × 4 in burnt plots and 3 × 2 in pristine plots). Different treatments were tested on different terraces to ease the leaching effect from adjacent conditions; in order to avoid contaminations among inoculated subplots, non-inoculated ones were set up at the top part of the hill.

Bioremediation treatments were designed to analyse *in situ* the role of indigenous microbiota and introduced microbes with or without plants on hydrocarbon removal and soil restoration. The exogenous microbes chosen were two wild-type strains of *Pseudomonas putida* harbouring the catabolic plasmid pWW0 (Ramos *et al*., 1991), the KT2440 (Bagdasarian *et al*., 1981) and BIRD-1 strains, being the latter recently reported to promote plant growth (Matilla *et al*., 2011; Roca *et al*., 2013). In order to stimulate the natural degradation of the pyrolytic compounds generated after fire, bioaugmentation of the indigenous hydrocarbon-degrading microbes was performed by isolating an indigenous hydrocarbon-degrading microbial consortium from the burnt soil by using diesel fuel as a carbon source, since it has a ≈ 25% of aromatic hydrocarbons (Agency for Substances and Disease Registry, http://www.atsdr.cdc.gov). Rhizoremediation assays were run with a combination of the described microorganisms and two kinds of plants, white clover (*Trifolium repens*) and Avex III**®** (Fertiprado), which is a commercial pasture seed mixture, composed of annual ryegrass, legumes and vetches and *Avena strigosa*. An organic solid vegetable support was used (commercial peat, COMPO**®**) as a carrier for microorganisms. Treatments applied are summarized in Table 1.

(i)  *Control:* The control bulk soil subplots remained untreated, providing an insight of the natural environment's recuperation capability; to be compared with any anthropic intervention made though the remediation treatments setting.
(ii)  *Control plants:* For each plot, 80 L of peat was homogeneously mixed with Avex III**®** seeds (10 g m^−2^) and *Trifolium repens* (clover) (5 g m^−2^) were spreaded over the soil surface, then, a slight topsoil work was made to achieve a homogeneous mixture of the peat with the soil and promote seed germination in the dark.
(iii)  *Bioremediation:* One litre of each microorganism/consortium (> 10^9^ cfu ml^−1^) was mixed with 10 L of tap water and then mixed with 80 L of peat. After spreading the mixture over the soil surface, topsoil was slightly worked to achieve a homogeneous mixture of the peat with the soil.
(iv)  *Rhizoremediation and treated pristine soil:* For each treated plot, 80 L of peat was homogeneously mixed with Avex III**®** (10 g m^−2^) and *Trifolium repens* (clover) (5 g m^−2^), then, 1 L of each microorganism/consortium (> 10^9^ cfu ml^−1^) was mixed with 10 L of tap water and then mixed with the peat-seed mixture. After spreading the mixture over the soil surface, the topsoil was slightly worked to achieve a homogeneous mixture of the peat with the soil and promote seed germination in the dark.

Characteristics that were assayed include bacterial survival, impact on indigenous microbial populations, soil quality parameters, hydrocarbon analysis and visual evolution of the landscape. For soil analyses, five subsamples per plot were collected from the upper 10 cm of topsoil and sieved at < 2 mm.

### Strains and culture media

The bacterial strains and plasmids used in this study are shown in Table 1. *Pseudomonas putida* BIRD-1 was grown in M9 minimal medium supplemented with sodium benzoate (10 mM) as the carbon source (Abril *et al*., 1989). The catabolic plasmid pWW0 was transferred to *P. putida* BIRD-1 by conjugation, as described by Ramos and colleagues (1991). *Pseudomonas putida* BIRD-1 harbouring the catabolic plasmid pWW0 was grown in M9 minimal medium supplemented with 3-methylbenzoate as carbon source, and supplemented with spectinomycin (100 μg ml^−1^) and rifampicin (20 μg ml^−1^). *Pseudomonas putida* KT2442R (pWW0) was grown in M9 minimal medium supplemented with toluene and rifampicin (10 μg ml^−1^). Cultures were incubated at 30°C and shaken on an orbital platform operating at 200 strokes per minute. Monitoring of survival of each strain in soil was performed by drop-plating dilution series in solid M9 minimal medium supplemented with the required carbon sources and antibiotics, as described previously.

Indigenous culturable microorganisms with the ability to degrade aromatic hydrocarbons were isolated by enrichment from the superficial layer of burnt soil (5 cm depth) using M9 minimal medium supplemented with diesel fuel as a carbon source. Monitoring of survival of soil microorganisms was performed by drop-plating dilution series in solid M9 minimal medium supplemented with diesel fuel in the vapour phase (100 μL per plate), as described above.

To perform bacterial plate count, rhizosphere soil was considered to be soil closely attached to roots sieved through a 2-mm mesh, and bulk soil was obtained from the 5–10 cm of topsoil and then also sieved. One gram of each soil sample was introduced in a Falcon tube with 9 ml of M9 medium and vortexed for 1 min to separate cells form soil particles and resuspend them. Then, dilution series were performed to obtain cfu per gram of soil.

For identity verification of the introduced *P. putida*, BIRD-1 and KT2440 strains, 100 bacterial colonies from each treatment (Treated pristine soil, Bioremediation and Rhizoremediation) were analyzed using REPc fingerprinting, as described by Aranda-Olmedo and colleagues (2002). Colony hybridization was also performed for 100 bacterial colonies per treatment, as described by Sambrook and colleagues (1989) using DNA probes corresponding to the *xylS* gene (to identify the pWW0 catabolic plasmid) or the PP_0314 gene (to identify *P. putida* KT2440); no colony hybridizations were performed for BIRD-1 because its genome was not sequenced at that time.

### Library construction and biodiversity analysis

Total DNA was extracted from approximately 0.5 g of a composed bulk soil sample from Control burnt bulk soil, from 0.5 g of a composed rhizospheric soil sample from Rhizoremediation treatment and from 0.5 g of a composed rhizospheric soil sample from Control pristine soil using the FastDNA kit (Qbiogene, Carlsbad, CA, USA) and purified on agarose gels. The universal Eubacterial primers GM3F (5′-AGAGTTTGATCMTGGC-3′) and GM4R (5′-TACCTTGTTACGACTT-3′) were used for amplifying the 16S rDNA gene (Muyzer *et al*., 1993). Each PCR reaction was performed in 50 μL reaction volume containing 5 μL of reaction buffer, 0.2 mM of primers, 2 mM MgCl_2_, 0.2 mM deoxynucleoside triphosphates, and 2.5 U DNA polymerase. The PCR conditions was as follows: 5 min of denaturation at 95°C, followed by 35 cycles of 1 min at 95°C, 1 min for primer annealing, 2 min at 72°C for primer extension, and a final cycle at 72°C for 10 min. The products of two consecutive PCRs were then pooled and purified through extraction from agarose gels prior to cloning into pGEM-T vectors. The resulting plasmids were transformed in competent *Escherichia coli* DH5α cells and positive transformants were colour-screened on LB plates supplemented with ampicillin (100 μg ml^−1^), Xgal (80 μg ml^−1^), and isopropyl-β-D-thiogalactopyranoside (20 mM). Clones with the correct insert were sequenced using the vector primers M13 F (5′-GGAAACAGCTATGACCATG-3′) and M13 R (5′-GTTGTAAAACGACGGCCAGT-3′).

The quality of the obtained sequences was manually checked using dna baser (http://www.dnabaser.com/download/download.html) and verified with bellerophon (Huber *et al*., 2004) and check_chimera (Maidak *et al*., 1996), and all chimeric sequences were discarded. These sequences were then compared with those in the GenBank database using the Blastn tool and the ribosomal database project database with classifier tool and aligned using clustalw (Thompson *et al*., 1994). DNA aligned of 16S rDNA gene sequences were used to construct a DNA distance matrix and rarefaction matrices with dotur package (Schloss and Handelsman, 2005). fastunifrac (Hamady *et al*., 2010) was used to produce PCoA comparing all samples.

### Hydrocarbons measurement

To perform the PAH analysis, soil samples were dried at 40°C and frozen. Defrosted samples were dried completely in a second step using an equivalent weight of mortar-ground anhydrous sodium sulfate. For PAH extraction, approximately 45 g of soil was placed inside a cellulose extraction thimble (Filtros ANOIA, Barcelona, Spain) and extracted with a mixture of dichloromethane:acetone (1:1) for 15 h. Once the extraction was completed, the organic solvents were evaporated and the remaining residue was re-dissolved in a small volume of dichloromethane (4–5 ml). To remove polar compounds, clean-up of the organic extract was performed using Sep-Pak® Plus Florisil cartridges (WATERS Corp., Milford, MA, USA), previously conditioned with 10 ml of dichloromethane. For the next step, dichloromethane was evaporated and the residue was resuspended in 2 ml of acetone. Finally, samples were filtered through a nylon Minisart syringe filter (0,45 μm, 13 mm Ø, Sartorius Stedim Biotech, GmbH, Goettingen, Germany). Analysis of PAH was carried out using an Agilent Technologies high-performance liquid chromatography system 1200 Series (Agilent Technologies, Santa Clara, CA, USA), equipped with a photodiode array detector (DAD, G1315D) and a scanning fluorescence detector (FLD, G1321A). The column used was a ZORBAX Eclipse PAH (Agilent Technologies, 5 μm, 4.6 I.D. × 150 mm). The mobile phase used was an acetonitrile-milli Q water gradient comprising 40% (v/v) acetonitrile from 0 to 1.25 min, programmed 100% (v/v) acetonitrile between 1.25 to 18 min. The initial solvent composition (60% milli Q water, 40% acetonitrile) was then maintained for further 3.5 min (Merck KGaA, Darmstadt, Germany).

Measurement of aromatic volatile organics, such as BTEX was performed by gas chromatography following EPA method 8020 (United States Environmental Protection Agency http://www.epa.gov).

### Measurement of soil quality parameters

#### Measurement of pH in soil

The pH values were measured in air-dried soil, sieved through 2 mm, using a glass combination electrode (soil: water ratio, 1:2.5 w:v), as described by Acosta-Martínez and colleagues (2003).

#### Measurement of phosphatase activity in soil

Phosphatase activity was determined as described by Antolín and colleagues (2005). The amount of 4-nitrophenol (PNP) released from 0.5 g soil, by triplicate, was measured after incubation, in the dark at 37°C for 2 h with 0.115 M 4-nitrophenyl phosphate-disodium (PNPP) as substrate for the enzymatic reaction, in 2 ml of maleate buffer (0.1 M, pH 6.5). Samples were cooled at 2°C for 15 min to stop the enzymatic reaction and 0.5 ml of 0.5 M CaCl_2_ and 2 ml of 0.5 M NaOH were added and well-mixed. Each sample was centrifuged at 2000 × *g* for 10 min. A blank experiment by duplicate was performed for each assay, in which the substrate was added to the soil sample after incubation and before stopping the reaction. The amount of PNP per hour released from each soil sample (μg PNP g^−1^h^−1^) was determined by comparing absorbance measures to a PNP standard curve. Rhizosphere soil was considered to be soil closely attached to roots, sieved through a 2 mm mesh.

#### Measurement of β-glucosidase activity in soil

β-glucosidase activity was determined as described by García and colleagues (1994). The amount of 4-nitrophenol (PNP) released from 0.5 g of soil, by triplicate, was measured after incubation in the dark, at 37°C for 2 h with 0.5 ml of 50 mM 4-nitrophenyl-β-D-glucopiranoside (PNG) as substrate for the enzymatic reaction, in 2 ml of maleate buffer (0.1 M, pH 6.5). Then, samples were cooled at 2°C for 15 min to stop the enzymatic reaction, and 0.5 ml of 0.5 M CaCl_2_ and 2 ml of 0.5 M NaOH were added and mixed well. Each sample was centrifuged at 3500 × *g* for 10 min. A blank experiment, by duplicate, was performed for each assay, in which the substrate was added to the soil sample after incubation and before stopping the reaction. The amount of PNP per hour released from each soil sample (μg PNP g^−1^h^−1^) was determined by comparing absorbance values to a PNP standard curve.

#### Measurement of DHA in soil

Dehydrogenase activity was determined as described by García and colleagues (1994). The amount of INTF released from 1 g soil, by triplicate, was measured after incubation in the dark, at 37°C for 20 h with 0.2 ml of 0.4% 2-(4-iodophenyl)-3-(4-nitrophenyl)-5-phenylteytrazolium chloride hydrate (INT) as substrate for the enzymatic reaction, and 2 ml of distilled water. Then, 5 ml of an extracting mixture (tetrachloroethylene: acetone (1:1.5 v:v) was added and well-mixed for 2 min. Each sample was centrifuged at 1000 × *g* for 10 min. A blank experiment, by duplicate, was performed for each assay, without substrate, in which the extracting mixture was added after incubation. The amount of INTF per hour released from each soil sample (μg INTF g^−1^h^−1^) was determined by comparing absorbance measures to an INTF standard curve.

#### Plant biomass monitoring

Five samples of clover and pasture plants were harvested from each plot at 4, 8 and 14 weeks sampling times. Plants were then manually separated into shoots and roots; fresh weight and length were recorded, and samples were dried in a stove at 90°C for 48 h. Samples were then allowed to cool to room temperature and dry weight was measured. The visual aspect of the area and each plot was photographed at each sampling time.

### Statistical analyses

A descriptive statistical analysis (the mean and absolute error) was calculated for each parameter. Also, we performed some inferential statistical analyses, such as analysis of variance (two-way analysis of variance) within treatments and soil type (burnt and non-burnt), assuming a normal distribution of the data and homoscedasticity. For post-hoc analysis, we used the Tukey test (*P* < 0.05) to determine changes in the analyzed parameters for each treatment.

## Conclusions

The use of Bioremediation and Rhizoremediation strategies in the current trial did not harm indigenous microbiota, and the release of non-native microbes only remained detectable in the soil for about 6 months after inoculation. Rhizoremediation treatments improved ecosystem resilience, accelerating its natural ability to return to the initial pre-fire state. The strains used in this study have proved their ability to survive in burnt soils, while exhibiting and a strong capacity to promote plant growth and development, making them suitable candidates for future use in the restoration of ecosystems affected by fires.

## Supporting Information

**Fig. S1.** Location of *Parque Natural de los Montes de Málaga* (green region) in the South of Spain. The red spot in the map corresponds to the location of the burnt site object of this study.

**Fig. S2.** Rarefaction analysis for pristine soil (P), bulk burnt soil (B) and rhizoremediation treatment (R) at two different times of the assay 1 month (autumn) and 6 months (spring). Rarefaction curves were constructed with DOTUR software.

**Fig. S3.** Introduced plants growth and soil coverage after 4 weeks of treatment on burnt soil. (A) shows non-inoculated plants and (B) shows plants on rhizoremediation treatment.

**Table S1.** Soil physic-chemical parameters for assessment of soil recovery, performed according to ORDEN 5/12/1975.
