# Supplementary figures and images for: Restoration of a Mediterranean forest after a fire: bioremediation and rhizoremediation field-scale trial
Source: Microb Biotechnol. 2014 Jul 31;8(1):77–92. doi: 10.1111/1751-7915.12138 (PMC4321375; doi:10.1111/1751-7915.12138)

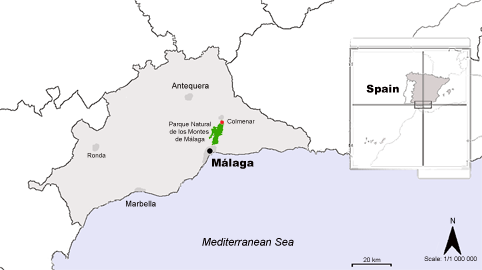

Supplement: Supplementary file 1 [file mbt20008-0077-sd1.tif]

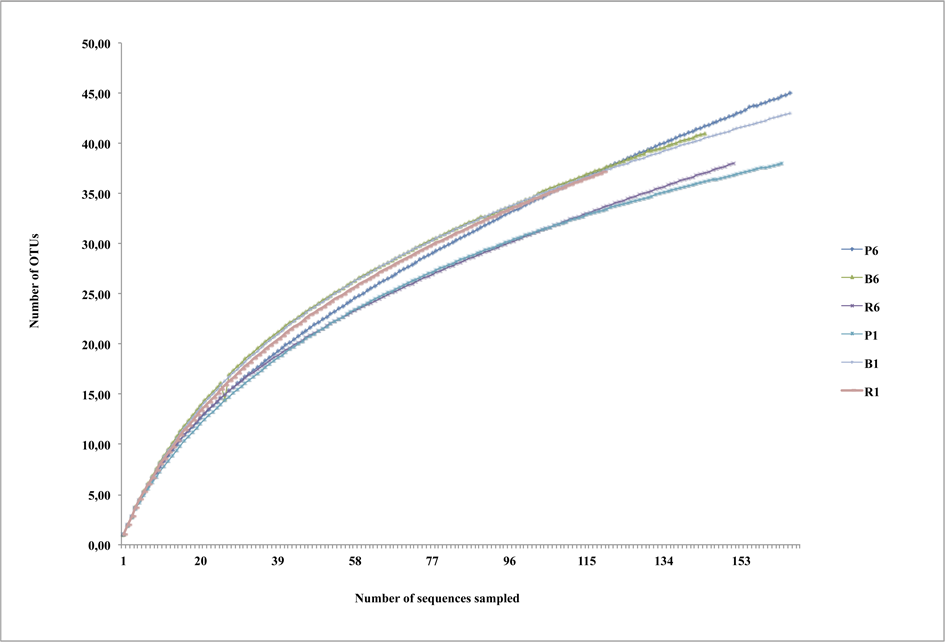

Supplement: Supplementary file 2 [file mbt20008-0077-sd2.tif]

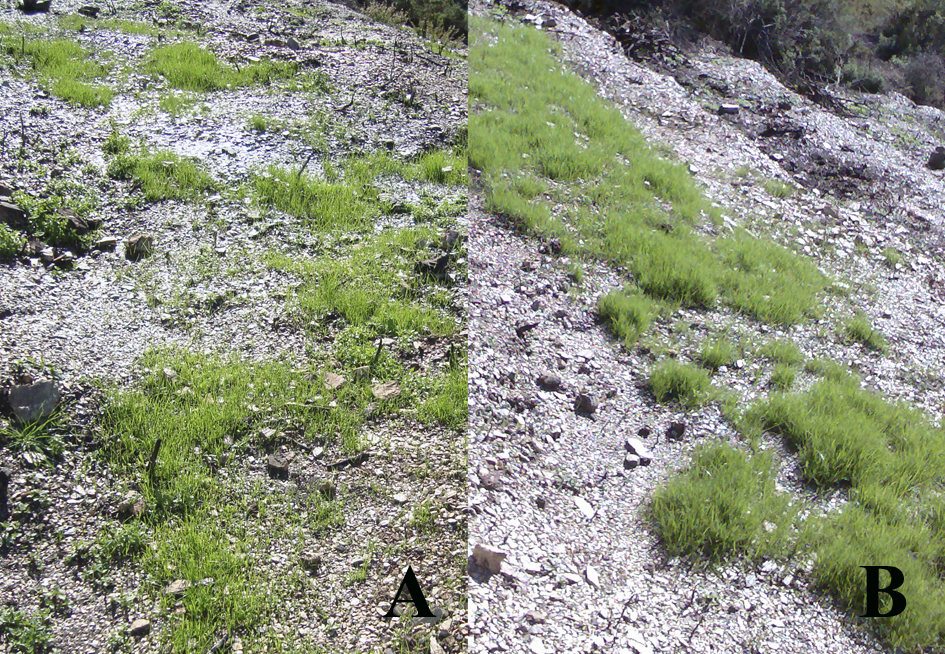

Supplement: Supplementary file 3 [file mbt20008-0077-sd3.tif]
