# Supplementary material for: Restoration of a Mediterranean forest after a fire: bioremediation and rhizoremediation field-scale trial
Source: Microb Biotechnol. 2014 Jul 31;8(1):77–92. doi: 10.1111/1751-7915.12138 (PMC4321375; doi:10.1111/1751-7915.12138)
Supplement: Supplementary file 4 [file mbt20008-0077-sd4.doc]

**Supplementary Table 1.** Soil physic-chemical parameters for assessment of soil recovery, performed according to ORDEN 5/12/1975.

| **Parameter** | **Control soil** | **Burnt soil** | **Soil after rhizoremediation treatment** |
| --- | --- | --- | --- |
| Organic matter (%) | 3.82 | 10.35 | 13.28 |
| Total N (%) | 0.189 | 0.457 | 0.7 |
| Available P (ppm) | 3 | 50 | 249 |
| Available K (ppm) | 100 | 490 | 250 |
| Salinity (mmhos/cm) | 0.15 | 0.50 | 0.86 |
